# Single‐Cell RNA Sequencing–Integrated Fibroblast Markers and Candidate Drug–Target Associations in Pancreatic Ductal Adenocarcinoma

**DOI:** 10.1155/humu/7172433

**Published:** 2026-11-06

**Authors:** Tengfei Ji, Hongsheng Wu, Zongmin Zheng, Keqiang Ma

**Affiliations:** ^1^ Department of Hepatobiliary Surgery The GuangZhou Affiliated Hospital of Jinan University (Huadu District People’s Hospital of Guangzhou, Huadu Institute of Medicine, Guangzhou) Guangzhou China

**Keywords:** cancer-associated fibroblasts, pancreatic ductal adenocarcinoma, single-cell RNA sequencing, survival-associated genes, weighted gene co-expression network analysis

## Abstract

**Background:**

Pancreatic ductal adenocarcinoma (PDAC) has dismal outcomes and is shaped by a fibroblast‐rich tumor microenvironment (TME). A cell type–resolved, module‐level understanding of cancer‐associated fibroblasts (CAFs) and their therapeutic relevance remains incompletely characterized.

**Methods:**

We integrated single‐cell RNA‐seq data from six treatment‐naïve PDAC resection specimens in GSE212966; individual tumor‐stage information was not available in the public metadata. Standardized preprocessing, clustering, and major‐cell‐type annotation were performed. Contact‐mediated intercellular signaling toward fibroblasts was profiled. High‐dimensional weighted gene co‐expression network analysis (hdWGCNA) was applied to fibroblasts to identify co‐expression modules and hub genes. Associations with overall survival were assessed by univariate Cox models and Kaplan–Meier analysis; immune infiltration was evaluated with CIBERSORT and ESTIMATE; pathway associations were examined by gene set enrichment analysis (GSEA). Candidate compounds were screened via Enrichr/DSigDB and docked to target proteins using a PyMOL–Chem3D–AutoDockTools workflow. SEMA3C was further examined in PDAC cell lines by siRNA‐mediated knockdown followed by qRT–PCR, CCK‐8, wound‐healing, and Transwell invasion assays.

**Results:**

We resolved 10 cell subpopulations and observed broad contact signals to fibroblasts, including the CD99–CD99 axis. hdWGCNA delineated fibroblast modules, from which four key modules were prioritized. Five genes—SEMA3C, PMP22, CPE, SNX9, and UACA—were significantly associated with overall survival. These biomarkers showed both positive and negative associations with specific immune‐cell fractions but were positively correlated with the StromalScore, ImmuneScore, and ESTIMATEScore. High‐expression groups were enriched for cancer‐relevant programs such as epithelial–mesenchymal transition, inflammatory response, and TNF*α*–NF‐*κ*B signaling, whereas several proliferative and metabolic pathways were enriched in the CPE‐low group. In vitro, siRNA‐mediated SEMA3C knockdown in PANC‐1 and Capan‐2 cells reduced cell viability, wound closure, and invasion, supporting a cell‐intrinsic protumorigenic role of SEMA3C in PDAC cells. Coumestrol emerged as a computationally prioritized compound, and molecular docking predicted a potentially favorable interaction with SNX9 (predicted binding energy −7.5 kcal/mol); this association requires experimental validation.

**Conclusion:**

An integrative single‐cell and network framework identified fibroblast‐related co‐expression modules and five survival‐associated genes in PDAC. The prognostic relevance of these genes and the predicted coumestrol–SNX9 association require further independent and experimental validation.

## 1. Introduction

More than 90% of pancreatic cancers are pancreatic ductal adenocarcinoma (PDAC), which is among the most lethal solid tumors [1–3]. The 5‐year survival rate remains in the single digits, and most patients present with unresectable or metastatic disease at diagnosis [4, 5]. Despite advances in perioperative management, chemotherapy regimens such as gemcitabine‐ or FOLFIRINOX‐based combinations, and a limited number of targeted and immunotherapeutic strategies, population‐level survival gains have been modest, with pervasive drug resistance and frequent relapse [6–8]. Beyond the intrinsic genomic instability and polyclonal evolution of malignant epithelial cells, the tumor microenvironment (TME)—characterized by profound immune suppression, a dense stromal barrier, and aberrant vasculature—has emerged as a major constraint on therapeutic efficacy [9–11]. Traditional tumor‐cell–centric paradigms therefore fail to account for the pronounced interpatient variability in treatment response and the rapid development of acquired resistance [12]. Reframing PDAC through the lens of tumor–microenvironment co‐evolution has become a central avenue for understanding disease biology and identifying new therapeutic entry points [13, 14]. In this context, systematically delineating the cellular composition, interactions, and molecular programs of the TME—and linking these features to clinical outcomes and treatment response—holds both scientific and translational value [15].

More than 90% of pancreatic cancers are pancreatic ductal adenocarcinoma (PDAC), which is among the most lethal solid tumors [1–3]. The 5‐year survival rate remains in the single digits, and most patients present with unresectable or metastatic disease at diagnosis [4, 5]. Despite advances in perioperative management, chemotherapy regimens such as gemcitabine‐ or FOLFIRINOX‐based combinations, and a limited number of targeted and immunotherapeutic strategies, population‐level survival gains have been modest, with pervasive drug resistance and frequent relapse [6–8]. Beyond the intrinsic genomic instability and polyclonal evolution of malignant epithelial cells, the tumor microenvironment (TME)—characterized by profound immune suppression, a dense stromal barrier, and aberrant vasculature—has emerged as a major constraint on therapeutic efficacy [9–11]. Traditional tumor‐cell–centric paradigms therefore fail to account for the pronounced interpatient variability in treatment response and the rapid development of acquired resistance [12]. Reframing PDAC through the lens of tumor–microenvironment co‐evolution has become a central avenue for understanding disease biology and identifying new therapeutic entry points [13, 14]. In this context, systematically delineating the cellular composition, interactions, and molecular programs of the TME—and linking these features to clinical outcomes and treatment response—holds both scientific and translational value [15].

Within the PDAC TME, cancer‐associated fibroblasts (CAFs) are abundant and functionally pivotal in shaping tumor ecology and determining therapeutic response [16–18]. By secreting and remodeling extracellular matrix (ECM), CAFs drive desmoplasia and tissue stiffening, alter interstitial pressure and fluid flow to create a drug‐delivery barrier, and, through cytokines, chemokines, and metabolites, reprogram angiogenesis and immune infiltration to foster immune suppression and escape [19, 20]. CAFs also act as “signal amplifiers” engaging in extensive ligand–receptor interactions with malignant, endothelial, and immune cells to coordinate tumor growth, invasion, and metastasis [21]. Importantly, CAFs are not a homogeneous population but display marked molecular and functional heterogeneity: Single‐cell and spatial omics studies have delineated myofibroblastic (myCAF), inflammatory (iCAF), and antigen‐presenting (apCAF) phenotypes that differ in spatial localization, secretory programs, immune regulation, and metabolic crosstalk [22, 23]. Multiple studies link CAF heterogeneity to patient survival, chemotherapy sensitivity, and responsiveness to immunotherapy [24, 25]; however, discrepancies persist across studies regarding subpopulation definitions, marker sets, and functional interpretations [26]. However, most previous studies have focused on predefined CAF subtypes or individual markers, whereas cell‐type‐resolved co‐expression modules linking fibroblast programs to prognosis and immune context in PDAC remain insufficiently characterized. Outside PDAC, CD99 has been detected in CAFs, and homophilic CD99 engagement has been shown to regulate *β*1‐integrin/FAK‐dependent cell adhesion [27, 28]. Nevertheless, whether CD99–CD99‐mediated contact influences CAF activation in PDAC remains unknown.

To address these gaps, we integrated a publicly available PDAC single‐cell dataset and applied high‐dimensional weighted gene co‐expression network analysis (hdWGCNA) to identify fibroblast‐related co‐expression modules at cellular resolution. We then prioritized module‐associated genes and assessed their relationships with survival, immune infiltration, and functional pathways in the TCGA‐PDAC cohort. In parallel, we combined drug‐perturbation resources with molecular docking to generate hypothesis‐level candidate compound–target associations. We hypothesized that fibroblast‐specific co‐expression analysis could identify clinically relevant stromal programs and generate experimentally testable hypotheses. Our contributions include a CAF‐centered, module‐level analytical framework for PDAC, five fibroblast‐related survival‐associated genes, and preliminary computational drug–target predictions requiring further validation.

## 2. Materials and Methods

### 2.1. Data Sources

Single‐cell RNA‐seq [29]. Public dataset GSE212966 was retrieved from GEO. The dataset contains six PDAC and six adjacent noncancerous resection specimens; only the six PDAC samples were included in the present analysis. All six PDAC specimens were obtained before treatment, whereas individual tumor‐stage information was not available in the public metadata. Sample‐level batch effects were addressed during data integration. All data were publicly available and de‐identified; no additional ethical approval was required.

TCGA‐PDAC cohort (TCGA‐PAAD). RNA‐seq expression data [30] and matched clinical information were obtained from the TCGA‐PAAD project through the GDC portal, extracting raw counts and FPKM values. We retained Primary Solid Tumor (01A) samples with overall survival (OS) ≥ 30 days, yielding 129 tumor cases for expression and prognostic analyses.

### 2.2. Preprocessing, Dimensionality Reduction, and Clustering of Single‐Cell RNA‐seq Data

Seurat was used to import single‐cell RNA‐seq count matrices and performed quality control by discarding cells with fewer than 200 or more than 6000 detected genes, retaining 49,191 cells for downstream analyses [31]. Counts were normalized and variance‐stabilized using SCTransform, followed by principal component analysis (PCA). To mitigate intersample batch effects, we applied Harmony to the PCA embeddings [32]. A k‐nearest neighbor (KNN) graph was then constructed from Euclidean distances in the top 50 principal components using FindNeighbors, and unsupervised clustering was performed with FindClusters (Louvain community detection; resolution = 0.05). Two‐dimensional embeddings were generated with UMAP for visualization. Cluster identities were assigned based on the expression of canonical marker genes curated from the CellMarker database, with manual curation using average‐expression profiles and marker distributions.

### 2.3. Differential Expression and Marker Identification Across Subpopulations

Differential expression was computed in a “one cluster versus all others” design using Seurat′s FindAllMarkers (default Wilcoxon rank‐sum test). Thresholds were set to logfc.threshold = 0.25 and min.pct = 0.30, with only.pos = TRUE to retain upregulated markers. Multiple testing was controlled using Seurat′s default Bonferroni correction (*p*_val_adj), and genes with *p*_val_adj < 0.05 were considered significant. For downstream analyses, markers were ranked by adjusted *p* value, average log fold change, and the proportion of expressing cells within each subcluster.

### 2.4. Functional Annotation of Cell Subpopulations

For each subpopulation, the upregulated (highly expressed) marker genes identified by differential analysis were subjected to functional annotation/enrichment using clusterProfiler to summarize the principal biological features and putative functions of each cluster [33].

### 2.5. Cell–Cell Communication Analysis

We inferred intercellular communication using CellChat and its curated ligand–receptor knowledgebase [34]. Treating fibroblasts as the receiver population, we extracted ligand–receptor interactions from other subpopulations to fibroblasts and restricted the analysis to the “Cell–Cell Contact” category. Interaction strength (communication probability) and statistical significance were estimated under CellChat′s default framework, with significance defined as adjusted *p* < 0.05. Bubble plots were used to visualize the probability and significance of intersubpopulation interactions.

### 2.6. High‐Dimensional WGCNA for Fibroblast‐Related Co‐Expression Modules and Hub Genes

We imported the single cell.rds object with hdWGCNA and subsetted the fibroblast population based on prior annotations [35]. A weighted co‐expression network was constructed on this subset. The soft‐thresholding power was selected at *β* = 8 using the scale‐free topology criterion, after which a signed adjacency matrix and topological overlap matrix (TOM) were computed. Hierarchical clustering with dynamic tree cutting was applied to detect co‐expression modules, yielding nine modules (M1–M9). For each module, we computed the module eigengene (ME) and intramodular connectivity/module membership and designated hub genes based on these connectivity metrics for downstream analyses.

### 2.7. Survival Association Analysis

Candidate genes were taken from the fibroblast‐related set identified by hdWGCNA. Tumor samples with complete survival information and a follow‐up duration (days) of ≥ 30 were included. OS was used as the endpoint (time unit: days; event = death; survivors were censored). For each gene, univariate Cox proportional hazards models were fitted with survival::coxph, treating normalized gene expression as a continuous covariate. We report the hazard ratio (HR), 95% confidence interval, and Wald test *p* value, and define *p* < 0.05 as statistically significant to designate survival‐associated genes. Kaplan–Meier curves were generated using survfit after dichotomizing patients into high and low expression groups using the optimal cutpoint determined with survminer::surv_cutpoint (minimum proportion per group = 0.20), and differences were assessed using the log‐rank test. The candidate genes were evaluated individually and were not combined into a multigene risk score or prognostic signature. The analyses were intended to identify fibroblast‐related genes associated with survival rather than to establish independent prognostic factors.

### 2.8. Immune Infiltration Assessment and Correlation Analyses

Immune‐cell composition was inferred using the CIBERSORT R implementation with the LM22 signature [36]. After obtaining the relative fractions of the 22 immune cell types for each sample, we evaluated associations between study biomarkers (gene expression or signature scores) and inferred infiltration levels using Spearman rank correlation, reporting the correlation coefficient (*ρ*) and *p* value; Benjamini–Hochberg adjustment was applied where multiple comparisons were made.

In addition, the estimate package was applied to the TCGA‐PDAC cohort to compute StromalScore, ImmuneScore, and ESTIMATEScore [37]. Associations between these scores and the biomarkers were assessed with Spearman correlation, with multiple‐testing correction applied as appropriate.

### 2.9. Gene Set Enrichment Analysis (GSEA)

Samples were dichotomized into high‐ and low‐expression groups based on the median expression of the target gene, and enrichment testing was performed with GSEA v4.4.0 under a binary phenotype design. The HALLMARK collection (MSigDB) was used as the gene set resource. Both positive and negative enrichment scores were examined to identify pathways enriched in the high‐ and low‐expression groups, respectively. An FDR *q* value < 0.05 was considered statistically significant.

### 2.10. CELL Culture and siRNA Transfection

The human pancreatic duct epithelial cell line HPDE6‐C7 (SCC442, Sigma‐Aldrich, Darmstadt, Germany) and the PDAC cell lines PANC‐1 (CRL‐1469, ATCC, Manassas, VA, United States) and Capan‐2 (HTB‐80, ATCC) were used in this study. Cells were cultured in Dulbecco′s modified Eagle′s medium (DMEM) supplemented with 10% fetal bovine serum and 1% penicillin–streptomycin at 37°C in a humidified atmosphere with 5% CO_2_, maintained as adherent monolayers and passaged with trypsin–EDTA when reaching 80%–90% confluence. Experiments were performed with cells in the logarithmic growth phase.

*SEMA3C* expression was silenced using small interfering RNAs targeting *SEMA3C* (si‐SEMA3C) and a nontargeting control siRNA (si‐NC). The sequences were si‐NC, 5 ^′^‐UUCUCCGAACGUGUCACGUTT‐3 ^′^; si‐SEMA3C#1, 5 ^′^‐GCGCUACUAAUUGGGAAGATT‐3 ^′^; and si‐SEMA3C#2, 5 ^′^‐GGGCUGAGGACCUUGCAGAAGATT‐3 ^′^. The siRNA oligonucleotides, including the nontargeting control, were synthesized by Shanghai GeneChem Co. Ltd. (Shanghai, China). PANC‐1 and Capan‐2 cells were seeded 24 h before transfection to reach 50%–60% confluence and transfected with 50‐nM siRNA using Lipofectamine 3000 (Thermo Fisher Scientific, United States) following the manufacturer′s instructions. Cells were collected at the indicated time points for RNA extraction and functional assays.

### 2.11. Quantitative Real‐Time PCR (qRT–PCR)

Total RNA was extracted from the cultured cells using TRIzol reagent (15596026CN, Invitrogen, Carlsbad, CA, United States). The amount and purity of the RNA were then assessed using a NanoDrop spectrophotometer (Thermo Fisher Scientific, United States). Next, first‐strand cDNA was created from the total RNA using a reverse transcription kit (D7178S, Beyotime, Shanghai, China). Finally, quantitative PCR was performed using SYBR Green qPCR Mix (D7260, Beyotime, China) on an Applied Biosystems real‐time PCR platform (Applied Biosystems, Foster City, CA, United States).

The amplification process began with a 10‐min denaturation step at 95°C, succeeded by 30 cycles of 15 s at 95°C and 30 s at 60°C, culminating in a 5‐min extension at 72°C. Relative mRNA levels were determined employing the 2^−*ΔΔ*Ct^ method, utilizing GAPDH as the reference gene. Primer sequences are presented in Table 1. Each sample underwent analysis in technical triplicate, and no‐template controls were incorporated into each experimental run.

**Table 1:** The primers utilized in this study.

Gene	Accession no.	Primers (5 ^′^‐3 ^′^)	
		Forward	Reverse
*SEMA3C*	NM_006379	ACCCACTGACTCAATGCAGAGG	CAGCCACTTGATAGATGCCTGC
*PMP22*	NM_000304	GGCAATGGACACGCAACTGATC	TGATCGACAGGATCATGGTGGC
*CPE*	NM_001873	TTGCTCCTGAGACCAAGGCTGT	GAGGAGCTGTATTCGTGAGCAC
*SNX9*	NM_016224	GGTCAGCCATTCCAATCCCTTC	ATGGCGACACATCCTGGTCATC
*UACA*	NM_018003	GGACACCACTTGTTCTGGCTAC	GCAACCTAGCATGAGGGCAGTT
*GAPDH*	NM_002046	GTCTCCTCTGACTTCAACAGCG	ACCACCCTGTTGCTGTAGCCAA

### 2.12. Cell Viability Assay

Cell viability was examined by plating cells in 96‐well plates at a density of 2 × 10^3^ cells per well. These cells were further cultured in DMEM medium containing 10% FBS and 1% penicillin–streptomycin. Cells were incubated at 37°C with 5% CO_2_, and cell viability was measured at 0, 24, 48, and 72 h after transfection. Viability was evaluated by Cell Counting Kit‐8 (CCK‐8; C0037, Beyotime). In each well, 10 *μ*L of CCK‐8 solution was added, and it was incubated for 4 h. Absorbance at 450 nm was then read with a microplate reader (Thermo Fisher Scientific). Relative viability was determined using the following equation: (OD_450_(sample) − OD_450_ (blank))/(OD_450_ (si‐NC) − OD_450_ (blank)) × 100%, normalized to si‐NC control. Three replicate measurements were analyzed for each experimental condition (*n* = 3).

### 2.13. Wound‐Healing Assay

Cells were plated in six‐well plates at approximately 5 × 10^5^ cells per well in 2 mL of complete medium and incubated at 37°C with 5% CO_2_ until approximately 90% confluence was reached. A straight scratch was then introduced using a sterile 200‐*μ*L pipette tip, and cell debris was removed by rinsing the wells twice with PBS. Then, serum‐free DMEM was added, and phase‐contrast images were acquired at 0 and 48 h using an inverted microscope. The wound‐healing rate was ascertained by calculating the percentage reduction in mean wound width at 48 h compared to the initial measurement. Each experimental condition was tested in triplicate before repeating the assay independently at least three times [38].

### 2.14. Transwell Invasion Assay

Cell invasion was evaluated using Transwell inserts with 8‐*μ*m pores. The upper surface of each insert was coated with Matrigel and incubated at 37°C for 30 min. A total of 3 × 10^4^ transfected cells suspended in serum‐free medium were added to the upper chamber, whereas medium containing serum was added to the lower chamber as a chemoattractant. After 48 h, noninvading cells remaining on the upper surface were gently removed. Cells that had invaded through the membrane were fixed with 4% paraformaldehyde for 15 min and stained with crystal violet for 10 min. Invaded cells were counted in at least five randomly selected microscopic fields per insert. Three replicate measurements were analyzed per condition.

### 2.15. Targeted Drug Prediction and Molecular Docking

The fibroblast‐related candidate genes were queried against DSigDB using the enrichR R package [39]. Hits with adjusted *p* < 0.05 were retained, entries explicitly restricted to specific cell lines were excluded, and nondrug chemical entities were removed by manual literature curation. Candidate small molecules were prioritized in ascending order of *p* value, and 3D ligand structures were downloaded from PubChem in SDF/3D format. The three‐dimensional structure of coumestrol was obtained from PubChem (CID: 5281707).

For receptor structures, target proteins were identified on UniProt, and cross‐referenced crystallographic entries were retrieved, preferentially selecting X‐ray structures with lower resolution values and greater sequence coverage. Molecular docking was used as a representative structural assessment rather than as a systematic druggability ranking of all candidate genes. Representative compound–target pairs were selected according to the availability and completeness of experimentally resolved receptor structures and the availability of compatible candidate ligands.

Structure preparation and docking proceeded as follows: crystallographic waters and cocrystallized small ligands were removed, and hydrogens were added in PyMOL; ligands were energy‐minimized in ChemBioOffice (Chem3D). Receptors and ligands were then prepared and docked using the AutoDockTools suite. For SNX9–coumestrol docking, the grid box was centered at *x* = 11.115, *y* = 43.771, and *z* = 48.921, with 126 × 126 × 126 grid points and a spacing of 1.0 Å. Docking poses were retained if the predicted binding energy was ≤ −5.0 kcal/mol and if at least one key ligand–receptor contact (e.g., hydrogen bond or hydrophobic interaction) lay within 3.5 Å. Resulting complexes were visually inspected and measured in PyMOL.

### 2.16. Statistical Analysis

Bioinformatic analyses were performed using R Version 4.3.0, and the Benjamini–Hochberg procedure was applied for multiple testing where appropriate, with an FDR *q* value < 0.05 considered statistically significant. In vitro data were analyzed using GraphPad Prism Version 10.6.1 and are presented as mean ± SD or SEM, as indicated in the figure legends (*n* = 3). Differences were assessed using two‐tailed unpaired Welch′s *t*‐tests or one‐ or two‐way ANOVA followed by appropriate multiple‐comparisons tests. *p* < 0.05 was considered statistically significant.

## 3. Results

### 3.1. Single‐Cell Atlas of PDAC

After quality control, normalization, batch correction, and dimensionality reduction/clustering, we resolved 10 major cell subpopulations in the UMAP embedding (Figure 1A–B). Using marker information curated from CellMarker 2.0 and pertinent literature, clusters were annotated as follows: T cells (*IL7R*, *CCL5*, *CD2*, *CD3D*); fibroblasts (*DCN*, *LUM*, *COL1A1*, *COL1A2*); macrophages (*C1QA*, *C1QB*, *HLA-DRA*); epithelial cells (*KRT19*, *MUC1*, *KRT8*); B cells (*IGHA1*, *CD79A*, *MS4A1*); smooth muscle cells (*MYH11*, *RGS5*, *ACTA2*, *MYL9*); endothelial cells (*VWF*, *EPAS1*, *COL4A1*); mast cells (*CPA3*, *CTSG*, *KIT*); Schwann cells (*S100B*, *CDH19*, *PLP1*); and MKI67^+^ progenitor‐like cells (*TOP2A*, *MKI67*). Marker expression patterns across clusters are summarized in a bubble plot (Figure 1C). Functional annotation of fibroblast‐specific upregulated genes revealed significant enrichment of ECM and matrix‐remodeling pathways (Figure 1D).

**Figure 1:**
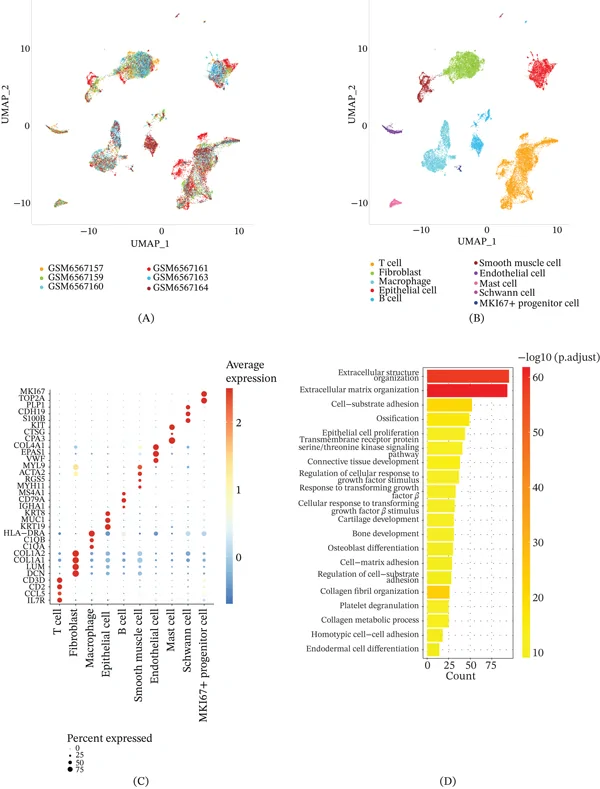
Single‐cell atlas of PDAC. (A) UMAP embedding colored by sample after batch correction. (B) UMAP embedding colored by cell type (10 subpopulations). (C) Bubble plot of canonical marker expression across clusters. (D) Functional annotation of fibroblast‐enriched genes.

### 3.2. Cell–Cell Communication Analysis

In multicellular systems, cellular functions depend on intercellular interactions, often mediated by multisubunit protein complexes. Our communication analysis revealed a widespread network of cross‐cluster signaling within the PDAC microenvironment. After extracting ligand–receptor pairs for each cluster and restricting to the Cell–Cell Contact category, predicted CD99–CD99 interactions were observed from T cells, macrophages, and epithelial cells to fibroblasts (Figure 2).

**Figure 2:**
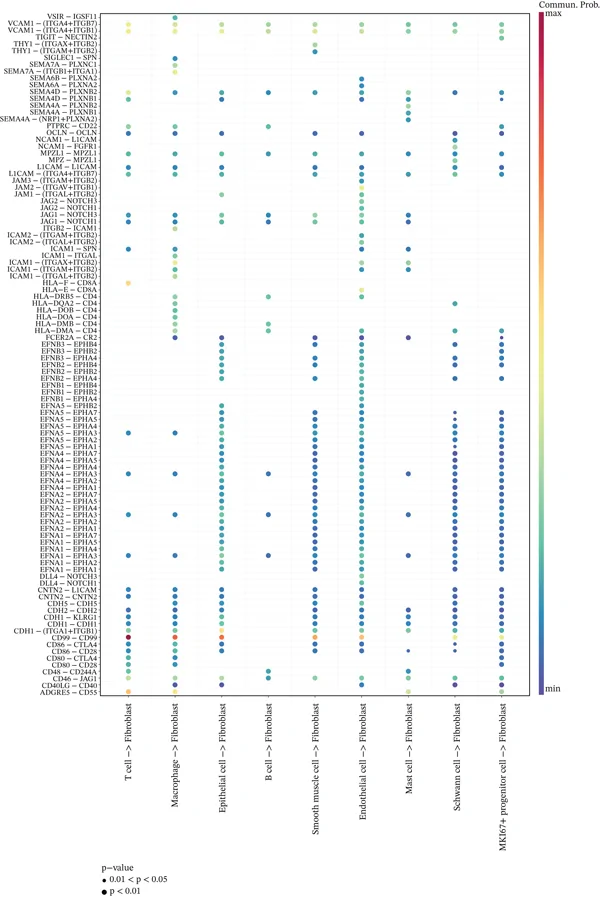
Ligand–receptor interactions targeting fibroblasts. Bubble plot of ligand–receptor interactions from all cell subpopulations to fibroblasts.

### 3.3. hdWGCNA Identifies Fibroblast‐Related Modules

The annotated single‐cell.rds file was imported into hdWGCNA, retaining genes expressed in ≥ 5% of cells to initialize the analysis object. To mitigate sparsity, metacells were generated using a KNN approach (*k* = 25) with the maximum number of shared cells per metacell pair set to 10. Weighted co‐expression analysis was then performed within the fibroblast subset: The soft‐thresholding power was automatically selected at *β* = 8 based on the scale‐free topology criterion (Figure 3A), from which a signed network and topological overlap were constructed (Figure 3B). Using MEs and intramodular connectivity, we prioritized hub genes and delineated nine robust modules (Figure 3C).

**Figure 3:**
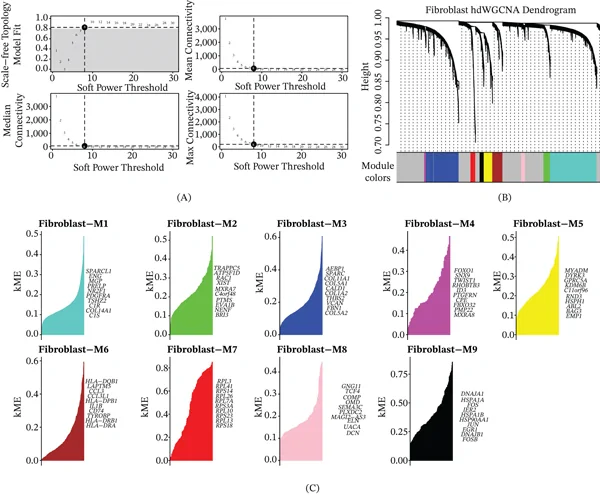
hdWGCNA‐based fibroblast module identification. (A) Scale‐free topology fit and connectivity across candidate soft‐thresholding powers. *β* = 8 was selected as the smallest power reaching a scale‐free topology fit of approximately *R* ^2^ = 0.80. (B) Co‐expression network dendrogram: upper panel, gene clustering tree; lower panel, module assignments. (C) Module delineation and hub genes: *y*‐axis shows kME (module membership) indicating connectivity to the module eigengene; right panel lists module hub genes.

We quantified, for each module, the proportion of expressing cells and the average expression across cell subpopulations and visualized intermodule correlations (Figure 4A). M1, M3, M4, and M8 exhibited higher expression prevalence and levels in fibroblasts (Figure 4B) and were therefore designated as key modules. Hub‐gene interaction networks were then extracted for these modules (Figure 4C–F): The inner ring (10 genes) denotes the primary hubs, whereas the outer ring (15 genes) represents secondary hubs, together delineating the core regulatory architecture within each module.

**Figure 4:**
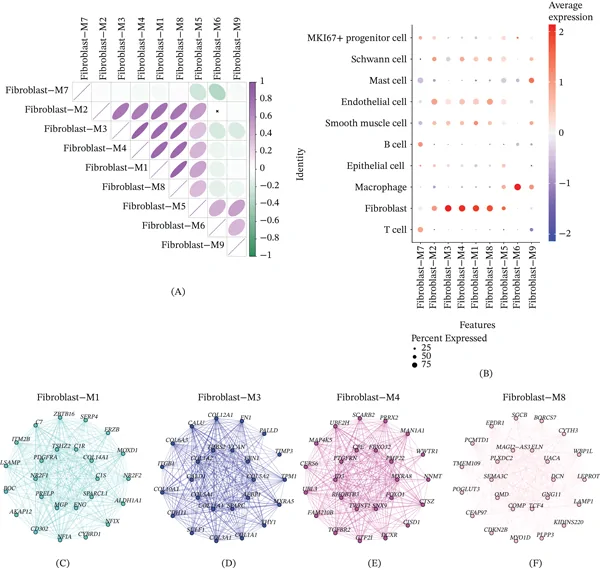
Key modules and hub‐gene networks. (A) Intermodule correlation matrix. (B) Expression of module genes across cell types; red = high, blue = low; dot size indicates the proportion of expressing cells. (C–F) Co‐expression networks of module hub genes; inner ring, primary hubs; outer ring, secondary hubs.

### 3.4. Survival Association Analysis

To assess the survival associations of fibroblast‐related genes derived from hdWGCNA, we fitted univariate Cox models in the TCGA‐PDAC cohort. Five genes—*SEMA3C*, *PMP22*, *CPE, SNX9*, and *UACA*—were significantly associated with OS (Figure 5A). Patients were then dichotomized into high‐ and low‐expression groups using survminer with optimal cutpoints (function surv_cutpoint; minimum proportion per group = 0.20) and analyzed by Kaplan–Meier methods. All five genes showed significant survival stratification (log‐rank *p* < 0.05; Figure 5B–F). These genes were evaluated as individual survival‐associated candidates and were not combined into a prognostic signature or risk‐score model.

Figure 5Survival association analysis of candidate genes. (A) Forest plot of univariate Cox models for survival‐associated genes. (B–F) Kaplan–Meier survival curves stratified by gene expression: (B) *CPE*, (C) *PMP22*, (D) *SEMA3C*, (E) *SNX9*, and (F) *UACA*.

**Figure:**
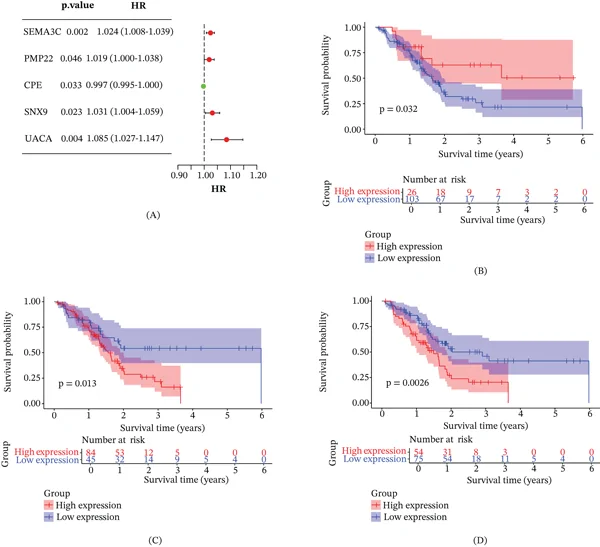

**Figure:**
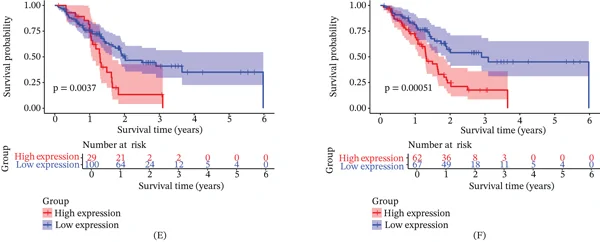

### 3.5. Correlations Between Biomarkers and Immune Infiltration

Using CIBERSORT–inferred proportions of 22 immune cell types, we computed Spearman correlations between the five biomarkers and immune infiltration levels and summarized the results in a correlation plot (Figure 6A). Multiple associations were statistically significant (adjusted *p* < 0.05), indicating associations with both innate and adaptive immune compartments. Consistently, ESTIMATE analysis revealed significant positive correlations of all five biomarkers with the StromalScore, ImmuneScore, and ESTIMATEScore (Figure 6B; Spearman *ρ* > 0, FDR < 0.05), supporting their association with a more stroma‐ and immune‐enriched microenvironment.

**Figure 6:**
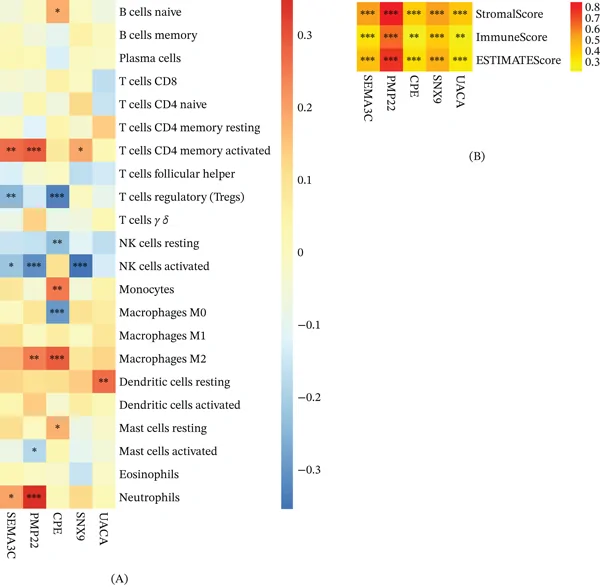
Correlations between biomarkers and immune infiltration. (A) Heatmap of correlations between the five biomarkers and the 22 immune cell fractions inferred by CIBERSORT. (B) Heatmap of correlations between the five biomarkers and ESTIMATE‐derived StromalScore, ImmuneScore, and ESTIMATEScore.  ^∗^Statistical significance is indicated as follows:  ^∗^ *p* < 0.05,  ^∗∗^ *p* < 0.01,  ^∗∗∗^ *p* < 0.001.

### 3.6. GSEA

To investigate the functional roles of the five biomarkers in tumors, samples were dichotomized at the median expression of each biomarker and analyzed using GSEA v4.4.0 under a binary phenotype design (FDR < 0.05). High‐expression groups showed significant enrichment of cancer‐related HALLMARK pathways, including epithelial–mesenchymal transition (EMT), inflammatory response, and TNF*α* signaling via NF‐*κ*B, among others (Figure 7A–E). Conversely, the CPE‐low group showed enrichment of E2F targets, MYC targets V1/V2, and oxidative phosphorylation, demonstrating that the analysis captured pathways associated with both high‐ and low‐expression phenotypes (Figure 7A).

**Figure 7:**
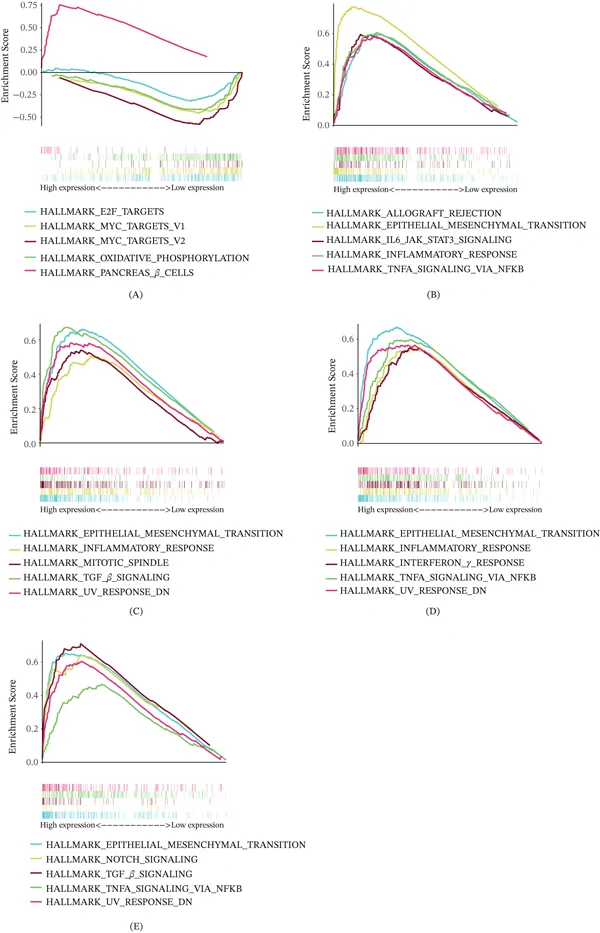
GSEA by biomarker expression. (A–E) GSEA plots for *CPE*, *PMP22*, *SEMA3C*, *SNX9*, and *UACA* (high vs. low expression). Upper panel shows the running enrichment score (ES); lower panels indicate the positions of gene‐set members along the ranked list. Positive enrichment scores indicate enrichment toward the high‐expression phenotype, whereas negative enrichment scores indicate enrichment toward the low‐expression phenotype.

### 3.7. Experimental Validation of SEMA3C in PDAC Cell Lines

The basal expression of the five fibroblast‐related candidate genes was first examined in a panel of pancreatic cell lines. qRT–PCR showed that SEMA3C, PMP22, CPE, SNX9, and UACA were expressed at higher levels in the PDAC cell lines PANC‐1 and Capan‐2 than in the nonmalignant pancreatic ductal epithelial cell line HPDE6‐C7, with SEMA3C showing one of the largest increases (Figure 8A). Two independent SEMA3C‐targeting siRNAs significantly reduced SEMA3C mRNA expression in PANC‐1 and Capan‐2 cells compared with si‐NC. Because si‐SEMA3C#1 produced the greater knockdown efficiency, it was selected for subsequent functional assays (Figure 8B,C). SEMA3C knockdown significantly reduced cell viability at 48 and 72 h (Figure 8D,E), decreased wound closure at 48 h (Figure 8F,H), and reduced the number of invading cells in the Matrigel‐coated Transwell assay (Figure 8G,I). These findings support a cell‐intrinsic role of SEMA3C in promoting PDAC‐cell proliferation, migration, and invasion; however, they do not establish a CAF‐specific function of SEMA3C.

**Figure 8:**
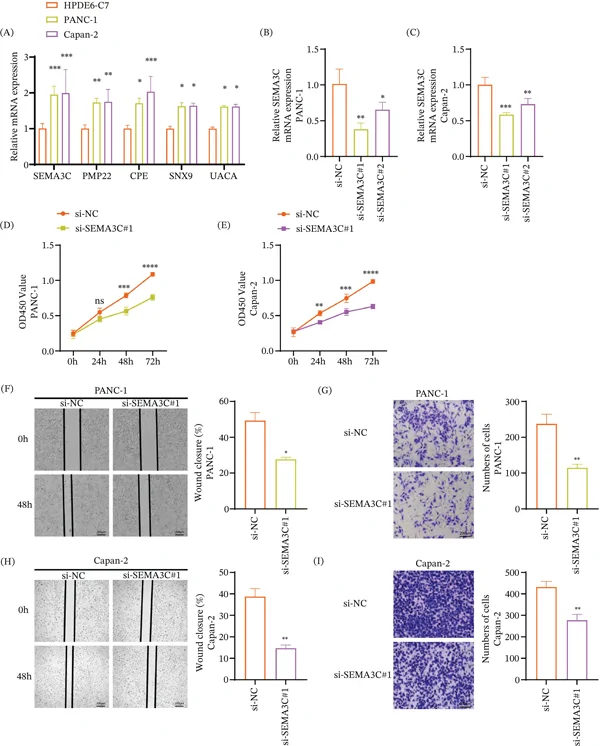
Experimental validation of SEMA3C in PDAC cells. (A) qRT–PCR analysis of *SEMA3C*, *PMP22*, *CPE*, *SNX9*, and *UACA* expression in HPDE6‐C7, PANC‐1, and Capan‐2 cells. (B, C) Knockdown efficiency of si‐SEMA3C#1 and si‐SEMA3C#2 compared with si‐NC in (B) PANC‐1 and (C) Capan‐2 cells. (D, E) CCK‐8 assays showing the effects of SEMA3C knockdown on cell viability in (D) PANC‐1 and (E) Capan‐2. (F, H) Wound‐healing assays in (F) PANC‐1 and (H) Capan‐2 cells transfected with si‐NC or si‐SEMA3C#1. (G, I) Matrigel‐coated Transwell invasion assays in (G) PANC‐1 and (I) Capan‐2 cells after si‐NC or si‐SEMA3C#1 transfection. Data are shown as mean ± SD.  ^∗^ *p* < 0.05,  ^∗∗^ *p* < 0.01,  ^∗∗∗^ *p* < 0.001,  ^∗∗∗∗^ *p* < 0.0001.

### 3.8. Drug Prioritization and Molecular Docking

Following DSigDB screening of the complete candidate‐gene set, the SNX9–coumestrol pair was selected for representative structural assessment because an experimentally resolved SNX9 structure (PDB: 3DYT) and a compatible candidate ligand were available. The predicted binding energy for the coumestrol–SNX9 complex was −7.5 kcal/mol, meeting the prespecified computational threshold of ≤ −5.0 kcal/mol and yielding a computationally favorable docking pose; predicted ligand–receptor contacts were within 3.5 Å (Figure 9). This selection was not intended as a systematic druggability ranking and does not indicate that the remaining four genes lack therapeutic potential. These docking results indicate structural compatibility in silico but do not establish direct molecular binding or pharmacological activity.

**Figure 9:**
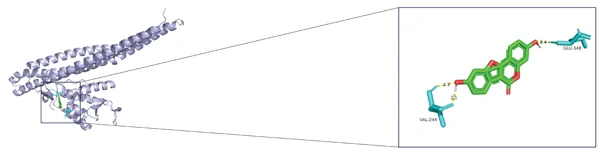
Predicted molecular docking pose of coumestrol with *SNX9*. Docking visualization: receptor protein in blue, ligand (coumestrol) in green, interacting residues in cyan; hydrogen bonds shown as yellow dashed lines with annotated distances in Å.

## 4. Discussion

PDAC is characterized by an exceedingly dense stroma and a profoundly immunosuppressive TME, in which CAFs are widely regarded as central drivers of fibrosis, immune evasion, and therapeutic resistance [40]. In this study, we profiled PDAC at the single‐cell and downstream network levels, showing that CAF‐upregulated genes are enriched in ECM and matrix‐remodeling pathways; intercellular communication analysis further suggested that immune/epithelial cells modulate CAF states through a CD99–CD99 contact axis. Within the fibroblast subset, hdWGCNA identified nine co‐expression modules and five fibroblast‐related genes associated with OS (*SEMA3C*, *PMP22*, *CPE*, *SNX9*, *UACA*). Collectively, these findings delineate a CAF‐centric framework that couples stromal remodeling with immune regulation and provide a roadmap for mechanistic studies and translational interventions.

CD99 is a homophilic cell‐surface adhesion molecule, and previous studies outside PDAC have detected CD99 expression in CAFs and linked CD99 engagement to the regulation of *β*1‐integrin/FAK‐dependent adhesion. In the present analysis, CD99–CD99 interactions were predicted between T cells, macrophages, epithelial cells, and fibroblasts. Such homophilic contacts could plausibly alter the duration or strength of immune/epithelial–fibroblast contact and thereby affect adhesion, cytoskeletal organization, and matrix‐remodeling programs associated with CAF states. Nevertheless, CellChat infers ligand–receptor compatibility from transcriptomic data and cannot determine signaling direction, CAF activation status, or causality. The proposed CD99‐dependent mechanism should therefore be regarded as hypothesis‐generating.

*SEMA3C* is upregulated in PDAC, where it promotes tumor‐cell survival and reshapes the immune milieu (including modulation of PD‐L1 and associations with M2‐like macrophage markers), and its high expression correlates with poorer survival [41]. Additional work links *SEMA3C* to tumor cell stemness/drug resistance and peritoneal metastasis, underscoring pleiotropic promalignant roles [42]. Consistent with these observations, our siRNA‐mediated knockdown experiments in PANC‐1 and Capan‐2 cells showed that SEMA3C depletion reduced cell viability, migration, and invasion in vitro, providing direct functional support for its protumorigenic role in PDAC. In parallel, CAF‐derived *SEMA3C* has been shown in colorectal cancer to drive liver metastasis via an NRP2–MAPK axis, supporting a stroma‐origin, paracrine mechanism and providing external support for its function in CAF contexts [43]. For *PMP22*, recent studies focusing on m6A regulation indicate that, in PDAC settings, *METTL14* can influence p53‐effector transcripts related to *PMP22* to promote proliferation and invasion, although direct, reproducible in vivo PDAC evidence remains limited [44]. *CPE* has been summarized across multiple cancers as a promoter of growth and metastasis; in PDAC cells, *CPE* knockdown suppresses proliferation, suggesting a protumor role, but direct data on its expression and function in CAFs are still lacking [45]. For *SNX9*, recent work demonstrates that deleting *SNX9* in T cells alleviates CD8^+^ T‐cell exhaustion and enhances CAR‐T efficacy, highlighting a key regulatory role on the immune side and implying that *SNX9* may affect PDAC outcomes via immune pathways [46]. *UACA* (Nucling) has been associated with inferior survival in recent transcriptome‐wide and TWAS analyses in breast and other solid tumors [47]; however, direct evidence in PDAC/CAF contexts remains insufficient and will require spatial and higher resolution validation. Importantly, PMP22, CPE, and UACA should not be interpreted as established PDAC CAF drivers. Their inclusion in the present study was based primarily on their membership in fibroblast‐derived co‐expression modules and their associations with survival and stromal scores. Thus, our results position these genes as module‐associated candidate markers rather than demonstrating fibroblast‐specific causal functions. Spatial localization and perturbation in defined CAF subsets will be necessary to determine whether they participate directly in PDAC stromal biology.

We observed mixed positive and negative correlations between the five genes and specific immune‐cell fractions, together with increased StromalScore, ImmuneScore, and ESTIMATEScore. For example, PMP22 and CPE were positively associated with M2 macrophages, whereas SEMA3C, PMP22, and SNX9 were negatively associated with activated NK cells. These findings suggest an immune‐enriched but potentially dysfunctional microenvironment rather than uniformly increased antitumor immunity. Dense CAF‐derived matrix may contribute to spatial T‐cell exclusion even when immune‐cell transcripts are detectable in bulk tissue. However, CIBERSORT estimates cell fractions but does not provide spatial information; therefore, the present correlations are not direct evidence of CAF‐mediated T‐cell exclusion. Mechanistically, *SEMA3C*′s regulation of PD‐L1 and association with M2‐like macrophage markers implicate a potential CAF/tumor‐cell–M2 macrophage–T‐cell suppression axis [48]. *SNX9*′s control of T‐cell exhaustion offers an immunologic lever that can help explain the concordance between high‐expression strata and worse outcomes [49]. At the microenvironmental level, CAFs can suppress T‐cell function via cytokine signaling, extracellular vesicles, and metabolic coupling, which aligns with our association patterns [50]. Moreover, the frequent activation of EMT and TNF*α*–NF‐*κ*B pathways in PDAC supports a self‐reinforcing loop linking matrix remodeling, inflammatory amplification, and immune suppression [51].

Recent in vitro and in vivo studies suggest that coumestrol exhibits antitumor activity across multiple malignancies, for example by inducing apoptosis and limiting invasion in colorectal cancer, with involvement of ferroptosis‐related pathways [52]. As a natural haspin inhibitor, a sprout‐enriched diet reduced intestinal polyps and improved cachexia‐related systemic phenotypes in mouse models, pointing to translational pharmacologic potential [53]. Epidemiologic analyses from PLCO further indicate that higher intake of total isoflavones and selected components (including coumestrol) associates with a lower risk of pancreatic cancer, predominantly among former smokers [54]. Overall, coumestrol remains at a preclinical/early‐evidence stage in oncology; logical next steps are to test it in PDAC models, particularly CAF coculture and immunocompetent systems, evaluate combination efficacy with standard regimens, and assess pharmacokinetics and safety while attending to potential endocrine and transporter‐related interactions. Importantly, epidemiological associations with dietary coumestrol intake do not demonstrate that physiologically achievable plasma or tumor‐tissue concentrations are sufficient to engage SNX9 or exert antitumor effects. Absorption, metabolism, protein binding, endocrine activity, and intratumoral exposure may differ substantially from concentrations used in pharmacological experiments. Therefore, our docking result should be interpreted only as a computational hypothesis rather than evidence of therapeutic efficacy.

This study nonetheless has limitations. First, the single‐cell analysis included only six treatment‐naïve PDAC resection specimens, and individual tumor‐stage information was unavailable. This limited sample size may not capture the full interpatient, stage‐related, and treatment‐related heterogeneity of CAF states. The survival analyses used gene‐specific Kaplan–Meier optimal cutpoints and univariable Cox models to screen individual survival‐associated candidates; no multigene risk score or clinical prediction model was constructed. Multivariable adjustment and independent external‐cohort validation were not performed. Therefore, the identified genes should not be interpreted as independent prognostic factors, and their survival associations require validation in future cohorts. The immune‐infiltration landscape was inferred by transcriptomic deconvolution and lacks spatial, imaging, or protein‐level confirmation. Future multiplex immunofluorescence or spatial‐transcriptomic studies should examine the colocalization of the five genes and CD99 with CAF markers such as FAP, ACTA2/*α*‐SMA, and PDGFRA, together with CD8‐positive T cells. The linkage between the five genes and CAFs is primarily based on transcriptomic and network associations; lineage specificity and causal functions require validation in defined myCAF, iCAF, and apCAF populations. The SEMA3C‐knockdown experiments in PANC‐1 and Capan‐2 cells demonstrate only a cancer‐cell‐intrinsic phenotype, and the absence of CAF–tumor‐cell coculture experiments prevents conclusions regarding paracrine or CAF‐specific SEMA3C functions. Finally, molecular docking provides predictive evidence only; direct binding assays, molecular‐dynamics simulations, cellular target‐engagement experiments, pharmacokinetic analyses, and in vivo efficacy and safety studies remain necessary.

## 5. Conclusion

This single‐cell–anchored study characterized the PDAC microenvironment and identified nine fibroblast co‐expression modules and five fibroblast‐related survival‐associated genes—SEMA3C, PMP22, CPE, SNX9, and UACA. Intercellular communication analysis identified a putative CD99–CD99 contact axis, whereas immune and pathway analyses linked the candidate genes to stromal enrichment and tumor‐related biological programs. SEMA3C knockdown reduced PDAC‐cell viability, migration, and invasion in vitro. Molecular docking further generated a preliminary coumestrol–SNX9 interaction hypothesis. Collectively, these findings provide candidate biomarkers and experimentally testable mechanisms, but their prognostic independence, CAF specificity, and pharmacological relevance require further validation.

**Nomenclature**
BH: Benjamini–Hochberg
CAF: cancer‐associated fibroblast
CD99: cluster of differentiation 99
CIBERSORT: Cell type Identification By Estimating Relative Subsets Of RNA Transcripts
CI: confidence interval
ECM: extracellular matrix
EMT: epithelial–mesenchymal transition
ESTIMATE: Estimation of Stromal and Immune cells in Malignant Tumor tissues using Expression
FDR: false discovery rate
FPKM: fragments per kilobase of transcript per million mapped reads
GSEA: gene set enrichment analysis
HALLMARK: MSigDB Hallmark gene‐set collection
hdWGCNA: high‐dimensional weighted gene co‐expression network analysis
HR: hazard ratio
KNN: k‐nearest neighbor
kME: module membership
LM22: leukocyte gene‐signature matrix of 22 immune cell types
MSigDB: Molecular Signatures Database
NF‐*κ*B: nuclear factor kappa‐B
OS: overall survival
PCA: principal component analysis
PDB: Protein Data Bank
PDAC: pancreatic ductal adenocarcinoma
QC: quality control
scRNA‐seq: single‐cell RNA sequencing
TCGA: The Cancer Genome Atlas
TME: tumor microenvironment
TOM: topological overlap matrix
UMAP: uniform manifold approximation and projection
WGCNA: weighted gene co‐expression network analysis

## Author Contributions

Tengfei Ji designed the study, performed data analysis, curated the dataset, drafted the original manuscript, and prepared visualizations. Hongsheng Wu and Zongmin Zheng assisted in data processing, validated results, and revised the manuscript. Keqiang Ma supervised the research, provided resources, and critically revised the manuscript.

## Funding

This project was supported by the Huadu District Basic and Applied Basic Research Joint Funding Project (District‐Hospital Collaboration), Guangzhou (No. 24HDQYLH010, Tengfei Ji) and the Key Medical Disciplines and Specialities Program of Guangzhou [2025‐2027].

## Ethics Statement

Ethical approval was not required for this study because it did not involve any human or animal experiments.

## Consent

The authors have nothing to report.

## Conflicts of Interest

The authors declare no conflicts of interest.

## Data Availability

The datasets generated and/or analyzed during the current study are available in the [GSE212966] repository, [https://www.ncbi.nlm.nih.gov/geo/query/acc.cgi?acc=GSE212966].
